# A novel laminin β gene *BmLanB1-w* regulates wing-specific cell adhesion in silkworm, *Bombyx mori*

**DOI:** 10.1038/srep12562

**Published:** 2015-07-27

**Authors:** Xiaoling Tong, Songzhen He, Jun Chen, Hai Hu, Zhonghuai Xiang, Cheng Lu, Fangyin Dai

**Affiliations:** 1 State Key Laboratory of Silkworm Genome Biology, Key Laboratory for Sericulture Functional Genomics and Biotechnology of Agricultural Ministry, Southwest University, Chongqing, 400700, China

## Abstract

Laminins are important basement membrane (BM) components with crucial roles in development. The numbers of laminin isoforms in various organisms are determined by the composition of the different α, β, and γ chains, and their coding genes, which are variable across spieces. In insects, only two α, one β, and one γ chains have been identified thus far. Here, we isolated a novel laminin β gene, *BmLanB1-w,* by positional cloning of the mutant (*crayfish*, *cf*) with blistered wings in silkworm. Gene structure analysis showed that a 2 bp deletion of the *BmLanB1-w* gene in the *cf* mutant caused a frame-shift in the open reading frame (ORF) and generated a premature stop codon. Knockdown of the *BmLanB1-w* gene produced individuals exhibiting blistered wings, indicating that this laminin gene was required for cell adhesion during wing development. We also identified laminin homologs in different species and showed that two copies of β laminin likely originated in Lepidoptera during evolution. Furthermore, phylogenetic and gene expression analyses of silkworm laminin genes revealed that the *BmLanB1-w* gene is newly evolved, and is required for wing-specific cell adhesion. This is the first report showing the tissue specific distribution and functional differentiation of β laminin in insects.

Laminins, a family of heterotrimeric glycoproteins, are the major biologically active components in basement membranes (BMs) and effectors of tissue architecture^1^,^2^. They are made of three different subunits, α, β, and γ chains, which assemble into a cruciform structure with three short arms and one long arm^2^. The short arms are associated with the polymerization of the molecule and the long arm is capable of binding to cellular receptors^3^. Thus, the laminins interact with other extracellular proteins and adhere to cells via receptors, such as integrins, dystroglycan, heparan sulfates, and sulfated glycolipids, thereby promoting adhesion, motility, proliferation, survival and differentiation of various cell types^4^,^5^,^6^.

Laminins are highly conserved across evolution, although the numbers of laminin isoforms and their coding genes are variable across spieces. In lower organisms, such as *Hydra*, there is only one laminin isoform, which is composed of one α, one β, and one γ gene^7^. Four laminin genes have been identified in protosomia (nematodes and insects) thus far. For example, it was discovered that the *Drosophila* genome contains two α genes, *wing blister* (*wb*, α1,2) and *laminin A* (*LanA*, α3,5), one β gene, *laminin B1* (*LanB1*), and one γ gene, *laminin B2* (*LanB2*).The four laminin genes are capable of assembling into two different heterotrimers. In complex organisms such as mammals, there are five α, four β, and three γ chains that give rise to at least 16 different isoforms^8^,^9^. It is presumed that the increase in the number of laminin genes through evolution could have occurred through a series of gene duplications and modifications.

Laminin isoforms differ in their composition of α, β, γ chains and may have cell- and tissue-specific distribution that reflects diverse biological functions. For example, in mammals, laminin 1, containing α1, β1, and γ1, has an essential function during early embryogenesis^10^, while laminin 2, made of α2, β1, and γ1 chains, is mainly expressed in muscle cells^11^. However, the laminin isoform composed of α3, β2, and γ3 chains, is essential for the CNS synaptic organization^12^.

Studies have suggested that the differential temporal and spatial distribution of laminins is mainly determined by variations in the expression of the α chain^9^. However, other studies have disproved this theory. In higher and lower species, absence or mutation in any laminin chain has resulted in embryonic lethality or severe disease condition that affects organ morphogenesis^7^,^13^,^14^. For instance, lack or partial loss of laminin α2 led to variation in skeletal muscle fibers and muscle fiber necrosis in mice and humans^15^,^16^. Mice with a mutation in laminin β1 or γ1 chain lack embryonic BMs and cannot survive past the late embryonic stage (day E5.5). In *Drosophila melanogaster*, subtle change in the *wb* gene causes a mild phenotype well known for wing blister, in which the dorsal and ventral wing surfaces separate. However, total lack of the gene results in early embryonic lethality^17^. *LanA* deficient mutants exhibit embryonic lethality with defects in the morphogenesis of heart, trachea and somatic muscle^18^. Besides, the elimination of LanB1 (β) prevents the normal morphogenesis of most organs and tissues, including the gut, trachea, muscles and nervous system, thereby resulting in mortality at the end of embryogenesis^19^. Thus, these evidences strongly support the notion that the spatio-temporal differentiation of laminins is due to all three chains.

In this study, we identified a new laminin β subunit gene (named *BmLanB1-w*) in silkworm by cloning *crayfish* (*cf*), a spontaneous recessive mutation with blister wing in homozygous mutants and no visible defect in any other tissues or stages. Previous studies have shown that in insecta, only one laminin β subunit is present, while we identified two β subunit genes in the silkworm genome. *BmLanB1-w* RNAi showed that the gene was essential for the interaction between the two chitinous wing layers in silk moths. Furthermore, the gene expression profile of silkworm laminin genes and phylogenetic analysis revealed that *BmLanB1-w* gene is a new member of the insect laminin β gene family and that it is abundantly expressed in the wing tissue.

## Results

### Phenotype analysis

**Figure 1:**
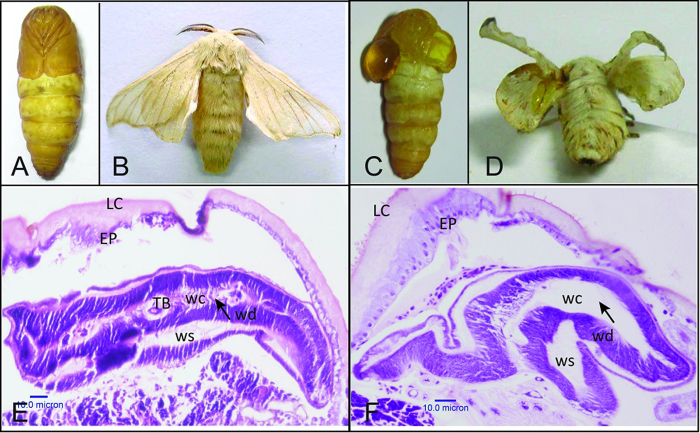
Phenotype of wild-type (*Dazao*) and *cf* mutant strain. Pupal and moth stages of wild-type (**A,B**) and *cf* (**C,D**) are shown. Hematoxylin–eosin stained paraffin sections of silkworm wing discs in late larval stage (day 6 of 5^th^ instar) of wild-type (**E**) and *cf* (**F**) are also shown. LC: larval cuticle, EP: epidermis, wc: wing cavity, ws: wing sac, wd: wing disc, TB: trachea tube.

The phenotypes of *cf* mutant (Fig. 1C,D) and wild-type (WT) (Fig. 1A,B) individuals were recorded using a digital camera (Canon EOS 5D Mark III). Wings of the *cf* mutant pupae are similar to the two large chelipeds in crayfish. Homozygous mutations of the *cf* locus led to one or two pairs of blister wings in the pupae and adult moths (Fig. 1C,D). The blistered pupal wings are filled with hemolymph between the dorsal and ventral layers. They are extremely fragile and can be easily wounded by slightly wobbling or touching, and result in bleeding to death.

To analyze how the wing discs of *cf* are different from the WT, the wing discs of both strains were dissected every day after the last larval molt. Visible differences were not observed in size and shape of the wing discs between the two strains. Further analysis of the inner structure of wing discs using paraffin sections revealed that in the WT the wing bilayer was attached all the time, and the trachea was located on the site of attachment (Fig. 1E). However, in the *cf* mutants the two wing disc layers failed to adhere to each other from late larval stage (day 5–6 in 5^th^ instar) (Fig. 1F). These results indicate that the *cf* defect occurred before larval-pupal metamorphosis, and that the phenotype was due to the separation of dorsal and ventral wing surfaces.

### Positional cloning of *cf* locus in *Bombyx mori*

**Figure 2:**
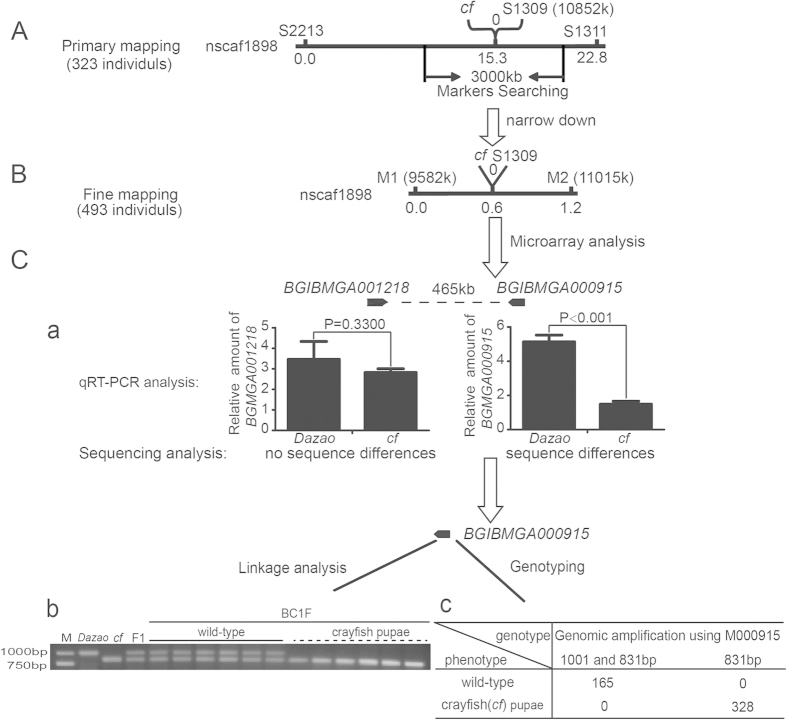
Mapping, bioinformatics and expression profile analysis of the *cf* locus. (**A**) Preliminary mapping of the *cf* locus. The *cf* locus was mapped between markers S2213 and S1311. The marker S1309 was tightly linked with the *cf* locus. Marker search in the genome was carried out within a 1500 kb sequence length both upstream and downstream of S1309. (**B**) Fine mapping of the *cf* locus. The *cf* locus was mapped between M1 and M2 using 493 BC_1_M individuals. S1309 was still tightly linked with the *cf* locus. (**C**) Screening the candidate gene and genotyping using polymorphism markers. Microarray analysis showed that there were only two genes (*BGIBMGA001218* and *BGIBMGA000915*) separated by a distance of 465 kb in the mapped region with different expression levels in the wild-type and the *cf* mutant. (**a**) qRT-PCR analysis of *BGIBMGA001218* revealed no significant difference in gene expression between the *cf* mutant and wild-type (The P value equals 0.3300, Student’s t-test. N = 3). Gene *BGIBMGA000915* within the mapped region was expressed much lower in *cf* than in wild-type based on both microarray and qRT-PCR analysis. Sequencing analysis of the two genes revealed that *BGIBMGA001218* was not different between the *cf* mutant and wild-type, but *BGIBMGA000915* differed. (**b**) Linkage analysis of the polymorphism marker M000915, which is 450 bp downstream of *BGIBMGA000915*. F1: first filial generation. BC1F: first backcross generation for linkage analysis. (**c**) Genotyping of the M000915 marker and *cf* locus using 493 BC1M (first backcross generation for recombination analysis) individuals. There is no recombination between M000915 and the *cf* locus. The results indicate that M000915 was also tightly linked with the *cf* locus.

To identify the candidate gene responsible for the *cf* phenotype, we performed primary mapping of the 323 BC_1_M progeny (a cross between *cf* ♀ × (*Dazao* × *cf*) ♂) using the SSR markers^20^. First, we roughly mapped the *cf* locus between the markers, S2213 and S1311, within scaffold nscaf1898 on the 13th chromosome. The distances from *cf* locus to these markers were 15.3 cM and 7.5 cM, respectively, and we found that marker S1309 was closely linked to the *cf* locus (Fig. 2A). We then designed new primer sets at 1500 kb upstream and downstream of S1309 flanking sequences based on the silkworm genome database. Genotyping using 493 BC_1_M individuals, further delimited the *cf* locus between markers M1 and M2, and the marker S1309 was still tightly linked to the *cf* locus (Fig. 2B). As a result, 63 genes were predicted to be present within this region (1433 kb).

**Figure 3:**
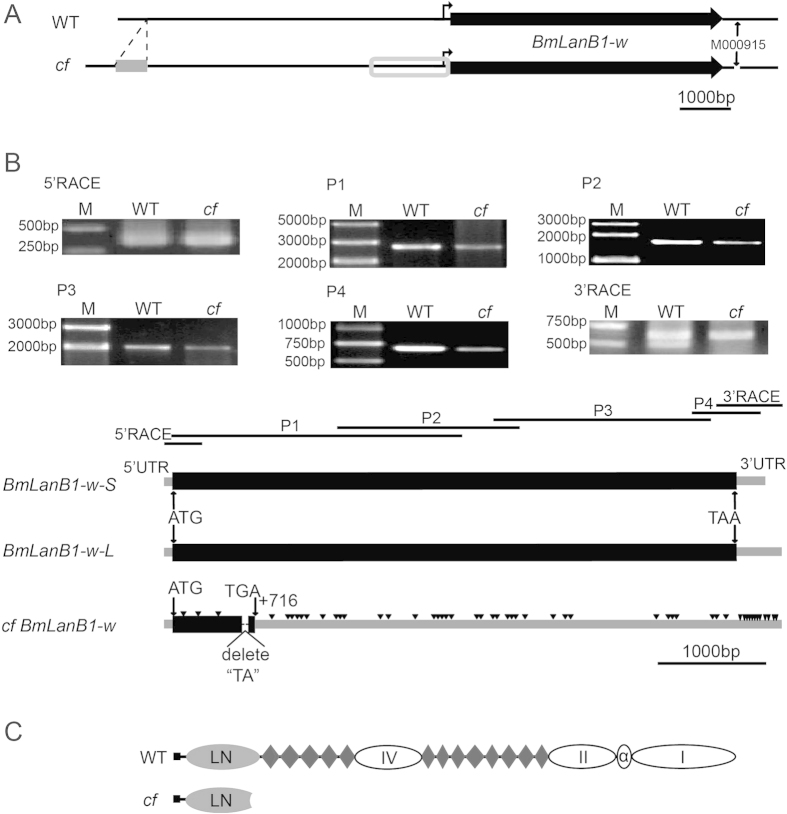
Schematic diagram of *BmLanB1-w* in wild-type (*Dazao*) and *cf* mutant. (**A**) Differences in the upstream and downstream sequences of *BmLanB1-w* between *Dazao* and *cf*. Gray rectangle indicates a non-LTR retrotransposon located 5.9 kb upstream of *BmLanB1-w* in the *cf* mutant. Gray box indicates the region upstream of *BmLanB1-w* in *cf* with sequences differing from *Dazao*. M000915 is a polymorphism marker 450 bp downstream of the *BGIBMGA000915* gene. Bold arrows indicate the location and transcriptional orientation of *BmLanB1-w*. (**B**) Cloning and characterization of the *BmLanB1-w* transcripts in *Dazao* and *cf* strains. We cloned the full-length cDNA sequences of the *BmLanB1-w* in *Dazao* and *cf* by RT-PCR and RACE techniques, using six primers (5′RACE, P1, P2, P3, P4, 3′RACE). The black horizontal lines indicate the location of the cloned fragments in *BmLanB1-w*, respectively. There are two types of transcripts (*BmLanB1-w-L* and *BmLanB1-w-S*) in *Dazao*, but only one type in *cf*. Black rectangles and gray bars indicate coding and untranslated regions of the *BmLanB1-w* exon, respectively. A 2 bp (“TA”) deletion was found in *BmLanB1-w* in the *cf* strain, and this deletion introduced a premature stop codon (TGA). Downward-pointing triangles represent nucleotide substitution in the *BmLanB1-w* in the *cf* strain. The scale bar corresponds to 1000 bp. (**C**) Predicted protein structure of *BmLanB1-w*. Gray oval represents LanB1 N-terminal domain, diamond represents laminin-type EGF-like domains, black rectangle represents signal peptide, and white oval represents other conserved domains.

To help identify the candidate gene for the *cf* phenotype, microarray analysis was performed using the wing discs of *Dazao* (WT) and *cf* at W0 stage (<5 hours after the start of larval wandering). The result showed that the expression levels of the two genes, *BGIBMGA001218* and *BGIBMGA000915*, in the narrowed region exhibited different patterns in the WT and the *cf* mutant (supplementary dataset). Using BLASTX search, we further found that *BGIBMGA001218* (nscaf1898:10187163…10194437) encoded a heat shock cognate protein, while, *BGIBMGA000915* (nscaf1898:10659797…10665195) encoded a laminin protein. To verify the microarray data, we performed qRT-PCR analysis on the genes within the mapped region. The results showed that only *BGIBMGA000915* had a significant change in expression level in the mutant wings (supplementary dataset). The expression levels of *BGIBMGA001218* varied considerably among individuals, and was not significantly different between the *cf* mutant and WT (The P value equals 0.33002) (Fig. 2C–a), Further sequences analysis of *BGIBMGA001218* revealed no sequence divergence between the *cf* mutant and WT silkworm. In *Drosophila*, the laminin homolog was reported to be essential for wing development, and a defect in laminin resulted in blistered wings^17^,^21^,^22^. Consistent with this finding in *Drosophila, BGIBMGA000915* was expressed at extremely lower level in the wing discs of the *cf* mutant when compared to the WT (Fig. 2C–a). Furthermore, genotyping revealed that another polymorphism marker, M000915, located at 450 bp downstream of the *BGIBMGA000915* termination codon (Fig. 3A) was tightly linked to the *cf* locus (Fig. 2C–b,c). Thus, we found that the *BGIBMGA000915* gene was located between these two closely linked markers, corresponding to 10852 k (S1309) and 10658 k (M000915), respectively in the nasca1898 scaffold. These results suggest that *BGIBMGA000915* was more likely the candidate gene for the *cf* phenotype. Based on the homologs in other organisms, we named it *BmLanB1-w.*

### Gene structure and expression analysis of the candidate gene

To clarify the structural features of the candidate gene, we cloned the full-length cDNA sequences of the *BmLanB1-w* in WT and *cf* by RT-PCR and RACE (rapid-amplification of cDNA ends) (Fig. 3A,B). Two types of transcripts, *BmLanB1-w-L* (5709 bp, Accession number: KP057212) and *BmLanB1-w-S* (5587 bp, Accession number: KP057213), were amplified from the wing discs of WT (Fig. 3B). Both had only one exon, which yielded the same *BmLanB1-w* protein (1779 amino acids) with a typical LanB1 N-terminal domain and 13 laminin-type EGF-like domains (Fig. 3C). However, only one *BmLanB1-w* (5707 bp) was detected in the *cf* mutant (Accession number: KP057211). We also examined the sequences upstream (~6 kb) of the transcription start site and found a non-LTR retrotransposon (560 bp) located 5.9 kb upstream of *BmLanB1-w* in the *cf* mutant (Fig. 3A, gray rectangle). In addition, there were a number of single nucleotide substitutions and short fragment insertions or deletions in the vicinity of the transcription start site (Fig. 3A, gray box) in the mutant. Compared to the *BmLanB1-w-L* coding sequence in the WT, *BmLanB1-w* in the *cf* mutant had 52 single nucleotide substitutions, and a 2 bp deletion, causing a frame-shift in the ORF and generating a premature stop codon (TGA) (Fig. 3B). The *cf*-type protein was predicted to contain a defective LanB1 N-terminal domain and lack all EGF-like domains (Fig. 3C). Multiple sequence alignments also suggested that the mutated protein most likely lacked the function of the WT protein.

**Figure 4:**
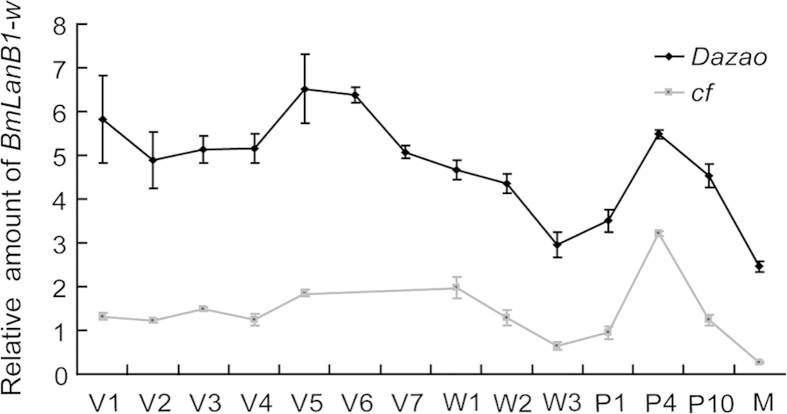
Temporal expression patterns of *BmLanB1-w* in *Dazao* and *cf* mutant. Quantitative RT-PCR analysis of *BmLanB1-w* was performed in wing discs and wings of wild-type (*Dazao*) and *cf* at different developmental stages. The *BmLanB1-w* gene expression had similar fluctuating patterns in both strains but was expressed at a significantly lower level in the *cf* mutant compared to the wild-type. All data are mean ± S.D (n = 3). V1-V7 represent 1st day to 7th of 5^th^ instar, respectively; W1-W3 represent day 1-day 3 of the wandering and spinning stages, respectively; P1, P4, P10 represent day 1, day 4, and day 10 of the pupae, respectively; M represents 1st day after eclosion. In wild-type (*Dazao*) the 5^th^ instar lasts 7 days, while in *cf* it is only 5 days.

Next, we compared the expression profiles of *BmLanB1-w* in the WT and *cf* mutant during wing morphogenesis by quantitative RT-PCR (qRT-PCR). In WT, the *BmLanB1-w* gene was expressed in the wing discs during all stages analyzed, from the 1^st^ day of the 5^th^ instar larval stage to the newly emerged moth. We observed two peaks; one at the end of the larval stage and the second at the mid-pupal stage, respectively. In contrast, expression of the *BmLanB1-w* gene had a fluctuating pattern that was similar between the *cf* mutant and the WT but the expression level was significantly lower in the *cf* mutant when compared to the WT (Fig. 4). Taken together, the predicted structural abnormality and the decreased expression level of *BmLanB1-w* in *cf* mutant strongly suggest that the *BmLanB1-w* gene was responsible for the *cf* phenotype.

### RNAi of *BmLanB1-w*

**Figure 5:**
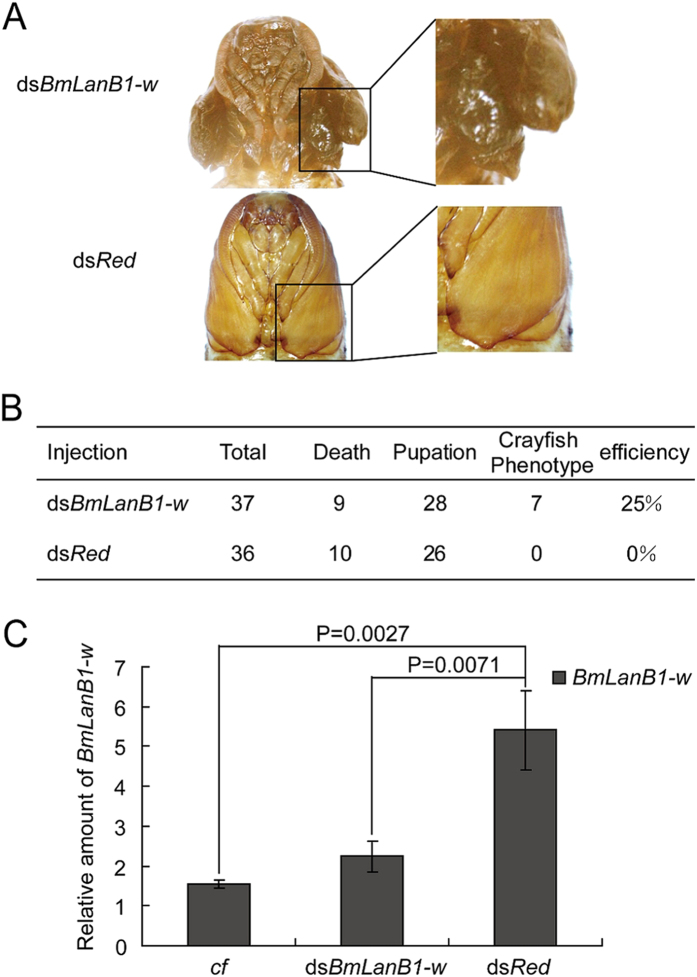
RNAi of *BmLanB1-w*. (**A**) Phenotypes of *BmLanB1-w* RNAi: wings with blisters were similar to wings in the *cf* mutant. Ds*Red* control did not show *cf* phenotype. (**B**) RNAi statistics. (**C**) Analysis of *BmLanB1-w* transcripts. All data are mean ± S.D (n = 3). Statistical analyses were performed using Student’s *t*-test (n = 3).

To verify whether the deficiency of *BmLanB1-w* could cause the blistered wing phenotype, we performed RNAi experiments to knockdown the expression of the gene in WT. We injected *BmLanB1-w* dsRNA (ds*BmLanB1-w*) twice into each 5^th^ instar larvae. The first injection was carried out at the beginning of wandering stage, and the second injection was performed 1 day later. The dsRNA of red fluorescent protein (ds*Red*) was used as a negative control. After injection, many larvae did not complete the larval-to-pupal metamorphosis but died because the bouble injections induced intractable wounds in the wandering larvae. The results obtained from the surviving larvae showed that similar to the *cf* mutant, 25% (7 of 28) of the WT individuals injected with ds*BmLanB1-w* developed wings with blisters after pupation (Fig. 5A,B). In contrast, there was no effect on the wing shape after injecting ds*Red* (n = 26). We then performed real-time PCR to investigate the expression level of *BmLanB1-w* after dsRNA injections. Compared to the negative control (ds*Red* injection), the level of *BmLanB1-w* transcription in the ds*BmLanB1-w* treated group was significantly lower and was as low as in the *cf* mutant (Fig. 5C). These results suggest that the blistered wing of *cf* is a result of *BmLanB1-w* loss of function.

### Laminin gene homologs in silkworm and phylogenetic analysis

As an essential regulator of morphogenesis of most organs, laminins require the simultaneous expression of all three chains and their assembly into α-β-γ heterotrimers to perform their roles in cell adhesion, migration, and rearrangement. In *Drosophila*, the elimination of *LanB1* prevents the normal morphogenesis of most organs and tissues, including the gut, trachea, muscles, and nervous system^19^. However, in the silkworm *cf* mutant, the *BmLanB1-w* defect did not affect any other tissue morphogenesis except the wings.

**Figure 6:**
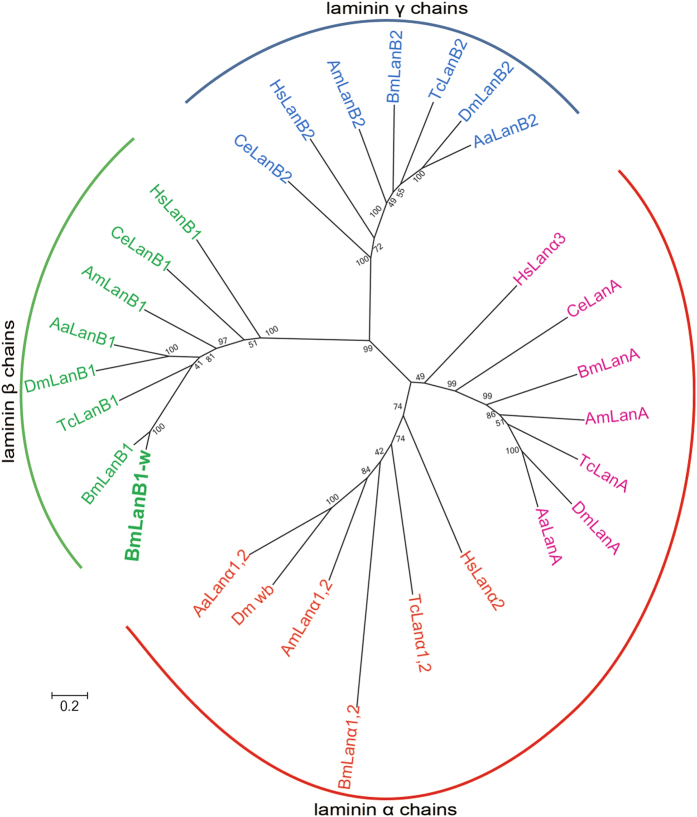
Phylogenetic analysis of laminins in various species. MEGA 6.0^24^ was used to construct the phylogenetic tree using the Maximum likelihood method. Numbers in the cladogram indicate bootstrap values. Abbreviations, Bm: *Bombyx mori*, Dm: *Drosophila melanogaster*, Am: *Apis mellifera*, Aa: *Aedes aegypti*, Tc: *Tribolium castaneum*, Hs: *Homo sapiens*, Ce: *Caenorhabditis elegans*. Accession numbers are listed in Table S2.

In order to investigate whether another *BmLanB1* gene in silkworm genome could compensate for the absence of the *BmLanB1-w* transcript, we identified all the laminin genes in silkworm by searching the conserved motifs in entire silkworm genome. We performed BLASTP and TBLASTN searches against the predicted silkworm gene in the Silkworm Genome Database, SilkDB (http://www.silkdb.org/silkdb/)^23^. We identified five laminin genes in the silkworm genome. To deduce the function and evolution of these laminin genes, we performed phylogenetic analysis of laminin proteins from different species by the Maximum likelihood method using MEGA6^24^. These laminin genes were also identified in the genomes of *C. elegans, H. sapiens* and four other insects including *D. melanogaster*, *A. aegypti*, *A. mellifera*, and *T. castaneum* by performing BLASTP and TBLASTN searches. The results were consistent with the laminin proteins in GenBank (http://www.ncbi.nih.gov/GenBank/). Therefore, we downloaded the amino acid sequences of the laminin subunits of the five insect species from GenBank for phylogenetic analysis. The resulting phylogenetic tree showed the division of laminins into three distinct clusters: laminin α chains, laminin β chains, laminin γ chains (Fig. 6). Based on sequence similarity, the five silkworm laminin genes were defined as two laminin α genes (*BmLanA*, *BmLanα1*, *2*), two laminin β genes (*BmLanB1-w*, *BmLanB1*), and one laminin γ gene (*BmLanB2*). It is noteworthy to mention that *BmLanB1-w* and *BmLanB1* shared 82.7% amino acid sequence identity and were grouped into one sub-clade, paralleling the other β chain genes in the subgroup. This indicates that the two laminin β genes were indeed present in the silkworm. To date, several laminin β paralogs have been reported only in the genome of Deuterostomia, and no additional β gene has been identified in lower Protostomia, which includes nematodes and insects. To investigate whether the duplication of laminin β also occurred in other lepidopteran species, we searched for the laminin genes in three other insect genomes, *P. xylostella*, *H. melpomene*, and *D. plexippus*, and compared the numbers of laminin genes in lepidopterans and other species (Fig. S1). As shown in the Fig. S1, the numbers of laminins are different among the four lepidoptera insects. *Plutella xylostella* has one additional α chain while silkworm has one extra β chain, suggesting that novel laminins originated independently in lepidopterans during evolution.

The high sequence similarity of two silkworm β subunits implied that they were generated through gene duplication. In order to examine the gene duplication events that generated new insect laminin genes, we used molecular phylogenetic analysis and compared gene contents between different species (Fig. S2). The phylogenetic tree was divided into two distinct orthologous groups; insects and vertebrates, respectively. In the insect clade, the two silkworm β genes formed a monophyletic subgroup and then clustered with each homolog in other insects. This suggested that the two silkworm laminin β genes were likely formed by the duplication within insects and could share a conserved function due to the high sequence similarity.

Together, these results support our hypothesis that in the *cf* mutant, another BmLanB1 chain probably compensated for the absence of BmLanB1-w chain in other organs and tissues except the wings.

### Spatial expression profile of silkworm laminin genes

**Figure 7:**
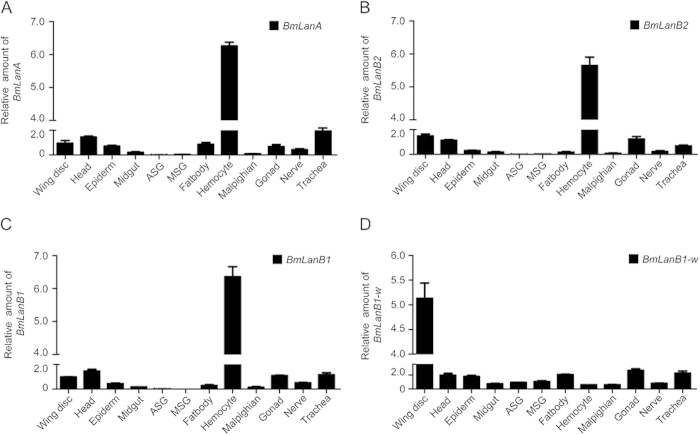
Spatial expression patterns of laminin genes in wild-type (*Dazao*). Quantitative RT-PCR analysis of laminin genes in 12 tissues in the 3^rd^ day of 5^th^ instar. The *BmLanB1-w* gene had robust expression in the wing discs and weak expression in other tissues. In contrast, the *BmLanB1* gene was expressed strongly in the hemocyte and weakly in wing discs and other organs, which was consistent with the expression patterns of laminin α (*BmLanA*) and laminin γ (*BmLanB2*) genes. Abbreviations, ASG: anterior silk glands, MSG: middle silk glands, Malpighian: malpighian tubule. The data indicate the mean ± S.D (n = 3).

Since two laminin β genes exist in the silkworm genome, we investigated their expression profiles in various tissues by qRT-PCR. Total RNA were extracted from 12 different tissues from day 3 of 5^th^ instar larvae. These included the wing disc, head, epidermis, midgut, fat body, hemocyte, malpighian tubule, gonad, trachea, anterior silk gland (ASG), middle silk gland (MSG), and nerve. The *BmLanB1* gene was expressed robustly in the hemocyte and at much lower levels in the wing disc, head, trachea, and other organs, which was consistent with the expression pattern of two other laminin subunits, laminin α gene (*BmLanA*) and laminin γ gene (*BmLanB2*). In contrast, the *BmLanB1-w* gene was transcribed much strongly in the wing discs and weakly in other tissues (Fig. 7). This comparison revealed the wing-specific abundance of *BmLanB1-w* and its importance in wing morphogenesis. Moreover, the co-expression of *BmLanB1*, *BmLanA*, and *BmLanB2* were likely sufficient to form α-β-γ heterotrimers in other tissues.

## Discussion

### *BmLanB1-w* is responsible for *cf* mutant

To identify the gene responsible for the *cf* mutant of silkworm, genetic mapping and microarray analysis were performed. The results showed that two genes, one heat shock cognate protein (Hsc70) gene (*BGIBMGA001218*) and one laminin beta gene (*BGIBMGA000915*), of the mapped region changed their expression levels in the *cf* mutant wings. In *Drosophila*, several heat shock proteins are involved in wing sheet adhesion. For example, Hsp83 RNAi was implicated in wing blistering, causing “swarovski” or “stump” wings^25^. The *BGIBMGA001218* gene is a homologue of a Hsc70 gene (FlyBase ID: *FBgn0026418*), and down-regulation of Hsc70-4 didn’t cause wing blisters in *D. melanogaster*^25^. The qRT-PCR analysis verified that only the *BGIBMGA000915* gene, but not the Hsc70 gene, significantly change its expression level in the mutant wings. The inaccurate description of the Hsc70 gene expression in the microarray analysis is because only one set of comparable samples were used, which didn’t detect the expression variation of this gene among individuals. Considering no significant differences in expression level and sequence of the heat shock cognate protein gene between *cf* mutant and WT, and no similar phenotype in *Drosophila* when its homologue was knocked down, we presumed that the “heat shock cognate protein” gene (*BGIBMGA001218*) might be not responsible for the *cf* mutant.

Then, we focused on the laminin beta gene, *BmLanB1-w,* of which homolog in *Drosophila* was essential for wing sheet adhesion. By cloning and comparing the sequences of the *BmLanB1-w* in WT and *cf*, a whole lot of aberrations in this gene were identified in the *cf* mutant, including nucleotide substitutions, frame shift, and retrotransposon insertion. Numerous aberrations in the related mutant gene is comprehensible, because the crayfish (*cf*) is a spontaneous mutant strain. Furthermore, it was expressed at extremely lower level in the wing discs of the *cf* mutant compared to WT, which might be caused by the non-LTR retrotransposon located in upstream of *BmLanB1-w* in *cf*, or by nonsense-mediated decay (NMD) of mRNA containing a premature stop codon as previous reports^26^,^27^,^28^,^29^, or by these two effects or some other reasons. RNAi experiments demonstrated that the deficiency of *BmLanB1-w* induced the blistered wing phenotype, suggesting that the *BmLanB1-w* is responsible for the *cf* mutant. This is the first report of a novel laminin β gene involved in wing-specific cell adhesion in silkworm, *Bombyx mori.*

### New laminin subunit in Insecta

Laminins are a large family of conserved proteins with crucial roles in development, adhesion and cell migration^30^. A number of studies have reported that the numbers of the laminin subunit genes increased from lower organisms (1 α, 1 β, and 1 γ chains) to higher organisms (5 α, 4 β, and 3 γ chains)^7^,^8^,^9^. However, only four laminin genes have been identified in insects thus far, including two α, one β, and one γ chains. In recent years, whole genome sequences from a variety of animal species have become available to identify laminins homologs in these species to better understand the evolution of laminins. Interestingly, we found that new laminin genes had emerged in lepidoptera insects. In the present study, a novel α chain gene and a novel β chain gene were identified in *P. xylostella* and *B. mori*, respectively. Phylogenetic analysis demonstrated that the laminin components in all lineages consistently had three subuints, suggesting that α, β and γ chains were the ancestral forms of laminin. The different subunits also have distinct functions, which are conserved during evolution. Lastly, the three ancestral laminin chains appear to have undergone multiple bifurcations during evolution to generate several paralogs by gene duplication. Taking the β chains as an example, the phylogenetic tree was divided into a vertebrate branch and an insect branch. In vertebrates, the β gene number increased to 3–6 copies. Lanβ1 and lanβ2 were clustered more closely in the phylogenetic tree, while, lanβ3 and lanβ4 belonged to another sub-group. Furthermore, evolutionary analysis suggested that lanβ1 and lanβ2 were generated by gene duplication before species divergence after which lanβ1 had duplicated again to yield lanβ1a and lanβ1b. Lanβ1a was absent in humans likely because of the loss of some chains as reported previously^31^. In contrast, lanβ3 and lanβ4 emerged by duplication after species formation. However, the two silkworm laminin β genes may have evolved from the same ancestral sequence by gene duplication during evolution of Lepidotera, probably within the silk moth lineage. Future sequencing of intermediate species may confirm such evolution. These results collectively indicate that laminin genes had undergone multiple independent duplications in evolutionary history. The duplication of laminin β during evolution of insects was independent of its duplication in vertebrates, such as *D. rerio* and *H. sapiens*. In summary, our data indicates that new laminin subunits evolved in insecta, likely due to an increased rate of evolution in this taxon, and the laminin β gene duplications occurred recently during evolution.

### Duplicated laminin β genes preserve original ancestral functio**n**

After gene duplications, the possible functions of the duplicated genes include conservation, neofunctionalization, subfunctionalization, and specialization^32^,^33^. In the present study, sequence analysis suggested that the two laminin β genes, *BmLanB1* and *BmLanB1-w*, were generated by gene duplication. This raised the question whether there is any functional diversity between the two genes.

*BmLanB1-w* gene was wing-specifically abundant during wing development. Its expression profile in the wing discs presented two peaks; one at the end of larval stage and another in the mid-pupal stage, respectively. The former expression peak may contribute to the elongation of wing discs and the association between the wing discs and the tracheal system. The later peak occurred simultaneously during vein formation, which suggests that it could improve vein development. This hypothesis was supported by observations in *Drosophila* that *LanB1* was expressed at the basal surface of veins from 32 to 44 h after pupation^19^. Furthermore, loss-of-function of the *BmLanB1-w* induced blistered wings, which demonstrated that *BmLanB1-w* was essential for cell adhesion between the dorsal and ventral wing surfaces. In *Drosophila*, flies carrying wing cell mutation for *LanB1* showed a strong wing blister phenotype consistent with the mutations disrupting the laminin α1,2 (*wb*) or laminin α3,5 chains (*LanA*)^17^,^21^,^34^. These similarities suggest that the *BmLanB1-w* gene had a conserved role in cell adhesion throughout evolution.

The next question was why are two laminin β genes required for silkworms? Deletion of *Drosophila LanB1* caused late embryonic lethality and defects in most organs and tissue morphogenesis^19^. In contrast, we found that *BmLanB1-w* was not required for the silkworm embryo and other tissues during development because no obvious defects were observed in the *cf* mutant or in individuals after *BmLanB1-w* knockdown. The spatial expression profile of different laminin genes in silkworm revealed that the expression pattern of *BmLanB1* was consistent with α and γ subunits in other tissues. Although *BmLanB1-w* was also detected in other organs, its expression level was not comparable with that in *BmLanB1* (data not shown). Therefore, it appears that *BmLanB1* is capable of compensating for the absence of *BmLanB1-w* transcript in other tissues but not in wings. In other words, the *BmLanB1* is required to fulfill the original function in border cells and tissues. Further, the co-expression of *BmLanB1* with other chains suggested that *BmLanB1* might be the ancestral copy, while the wing-specific abundance and the effect of *BmLanB1-w* gene suggested that it could be the newly generated copy by gene duplication. From these observations, we presume that the ancestral silkworm laminin β functions had undergone tissue differentiation. The newly evolved *BmLanB1-w* was necessary for the wing-specific cell adhesion, and both *BmLanB1* and *BmLanB1-w* were required to preserve all ancestral functions.

### Tissue-specific cell adhesion of laminins

The expression patterns of laminin isoforms are often tissue- and temporal-specific during development^35^. However, the underlying molecular mechanisms regulating their expression still remain unknown. Many previous studies suggested that the differential expression is mainly (but not exclusively) determined by variations in the expression of α chains^9^. For example, in mammals, the α1 chain is highly expressed in the epithelia of early embryos, and in adult reproductive organs including kidney and liver^36^. The α2 chain is mostly localized in the neuromuscular system^37^. The α3 chain is mainly found in the skin and other epithelia^38^,^39^. The reason for the tissue specific expression of α is presumed to be due to the large size of this chain, which contains the major cell-adhesive sites. For example, its long arm on the C-terminal end consists the LG1–5 domains, which are involved in the interactions with cellular receptors such as integrins and dystroglycans. The N-terminal end of the short arm is also capable of binding to integrin receptors^40^. Here, we demonstrated that the tissue-specificity of laminins could also be determined by the β chain, which contains two C-terminal globular domains that may also link the laminin molecules to the cell surface through direct or indirect interactions with cellular receptors, such as integrin, collagen, and sulfated glycolipids. This hypothesis is supported by reports that the β3 chain of laminin 5 binds to collagen VII^41^, and that the β2 chain could modulate integrin binding affinities of laminins^42^. An alternative explanation is that some cis-regulatory elements of laminin gene could regulate the spatiotemporal expression.

### Mechanism leading to *cf* mutation

**Figure 8:**
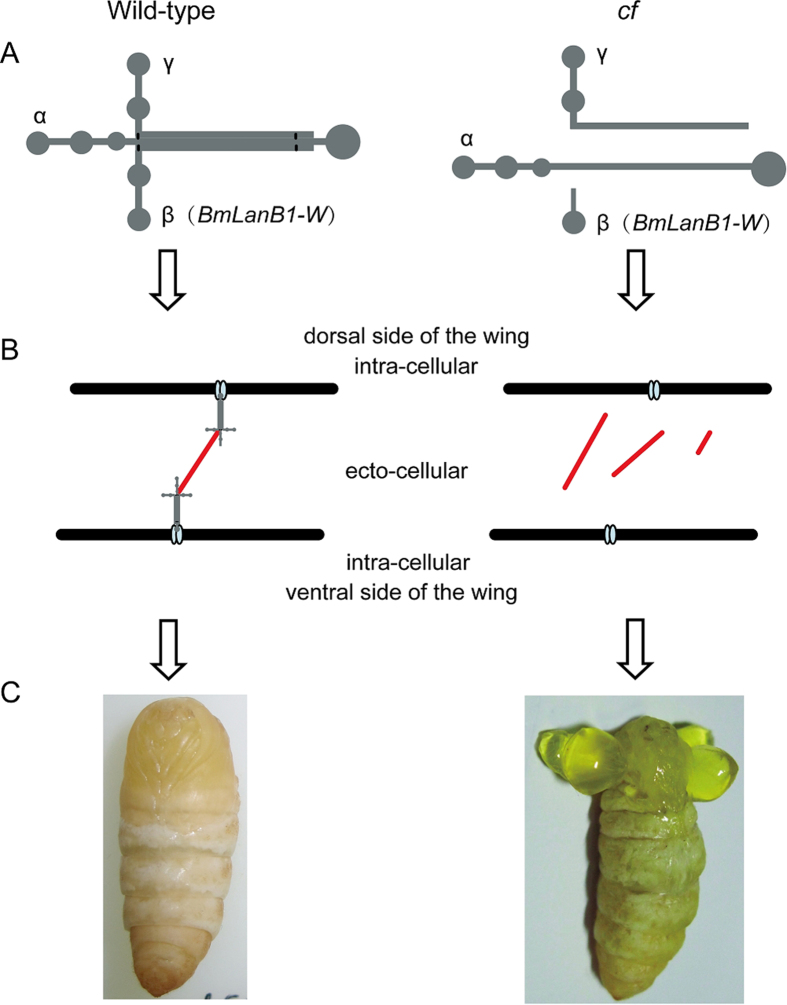
Schematic representation of the mechanism leading to *cf* phenotype. (**A**) Laminin structure in wild-type and *cf* mutant. In the *cf* mutant, laminin trimer cannot form due to the defect in *BmLanB1-w*. (**B**) Model diagram depicting the adhesion of dorsal and ventral sides of the wings in wild-type and *cf.* In the *cf* mutant, dorsal and ventral sides of the wings are not linked because normal basement membrane cannot form due to dysfunctional laminin trimer, which does not allow wings to withstand the hemolymph pressure thus resulting in wing blisters. Light blue oval represents integrins, gray pattern represents laminin trimer, red line indicates proteins such as collagen IV that are linked to the laminin trimer to enable basement membrane assembly. (**C**) Phenotypes of wild-type and *cf*.

Based on our findings we predict the following mechanism that leads to *cf* mutation. The wing is composed of two epithelial sheets that adhere tightly to each other. Laminin is the secreted protein localized in the extracellular basal membrane of the epidermis^43^. In the wild type imaginal wing discs, the β chain encoded by *BmLanB1-w* gene is assembled with α and γ chains to form the heterotrimeric laminin. Then, heterotimers are self-assembled into a cell-associated network through the ternary nodes, which is formed by the common domain structure in the short arms of each chain. Finally, polymerized laminins bind to the cell surface through interactions with cellular receptors, such as collagen and integrin, making dorsal and ventral sides of wings attached together thus enabling normal wings to withstand the pressure of the hemolymph (Fig. 8). However, in the *cf* mutant, ablation of the BmLanB1-w chain in wings prevents laminin heterotrimer formation and basement membrane assembly, causing a failure in adhesion between the two layers of the wing. The wings are filled with hemolymph between the two sheets, eventually forming the *cf* blistered wings phenotype (Fig. 8).

In conclusion, our results demonstrated that loss-of-function of a novel laminin family gene, *BmLanB1-w*, is responsible for the *cf* phenotype with blistered wings in *B. mori*. We also showed the existence of two laminin β genes in silkworm. This is the first report of two laminin β subunits in insecta. The spatial expression profile analysis suggested that the two genes might perform sub-functions of the ancestral gene and may have diverged in a spatial pattern. *BmLanB1* maintained the function in a broad range of tissues, while the new laminin gene was evolved to perform tissue specific functions in cellular adhesion during wing organogenesis. Further studies are needed to address how laminins can perform such different functions during development. The information derived from these studies should advance our understanding in the evolutionary history and functional differentiation of laminin genes.

## Materials and Methods

### Silkworm Strains

The *crayfish* mutant (*cf*) and the *Dazao* wild-type (WT) silkworm were provided by the Silkworm Gene Bank in Southwest University, China. The silkworms were reared on fresh mulberry leaves under a 12 h/12 h light/dark photoperiod at 25 °C during the experiments.

### Paraffin section of silkworm wing discs

To detect the phenotype differences in the wing discs of WT (*Dazao*) and *cf*, the anterior and posterior wing discs were dissected from the two strains every day after the last larval molt. First, we cut the integument near the wing discs with a razor blade. Then, wing discs were extracted carefully, fixed in Bouin’s fixative at room temperature for 24 h, dehydrated first in a graded series of ethanol followed by a series of xylene, and eventually embedded in paraffin wax. A series of continuous sections (5 μm) were obtained using a LEICA RM2235 microtome (Germany) and stained with hematoxylin-eosin using standard protocols^44^. Subsequently, sections were observed through a Nikon C-DSS230 stereomicroscope (Japan).

### Mapping of *cf* Locus

Two silkworm strains, *Dazao* (+^*cf*^/+^*cf*^) and *cf (cf*/*cf*), were selected as parents for genetic mapping. F1 offspring were obtained from the cross between female WT (*Dazao*) and male *cf* mutant. For linkage analysis, 20 BC_1_F progeny (10 WT and 10 mutant) from the cross (*Dazao* × *cf*) ♀ × *cf*♂ were used. For recombination analysis, the BC_1_M offspring of *cf*♀ × (*Dazao* × *cf*) ♂ were used. SSR markers from the published SSR molecular linkage map were used for preliminary mapping. Based on the preliminary mapping results, we analyzed sequences both upstream and downstream of the SSR marker that were tightly linked with the *cf* locus. New primers were designed based on the sequences adjacent to the predicted genes, and markers that exhibited polymorphism between parents were used to genotype the 493 BC_1_M progeny. For this analysis, we used the silkworm 9 × assembly genome database (http://www.silkdb.org/silkdb/), BLAST^45^, and primer 5.0. The primers used for mapping are listed in Table S1.

### Microarray Analysis

Total RNA from the wing discs of *Dazao* and *cf* at W0 (<5 h after the start of larval wandering) were extracted using EZ.N.A^TM^ Microelute Total RNA Kit (Omega). Microarray was performed by CapitalBio Corporation (Beijing, China). Microarray data analysis was performed as described previously^46^.

### cDNA sequence cloning

Total RNA were extracted from the wing discs of *Dazao* and *cf* at W0. Primers were designed based on the sequences of the predicted genes (*BGIBMGA000915* and *BGIBMGA001218*) from silkDB (http://www.silkdb.org/silkdb/). Full-length cDNA of the genes was obtained using GeneRacer^TM^ Kit (Invitrogen) according to the manufacturer’s protocol. PCR products were cloned into the pMD19-T vector (Takara) for sequencing. Primers used for cloning are listed in Table S1.

### Quantitative RT-PCR

ABI Prism7000 sequence detecting system (Applied Biosystems, Foster City, CA, USA) and SYBR Premix EX Taq-kit (Takara) were used for qRT-PCR to investigate the expression levels of the candidate gene in the wing discs at different developmental stages (from 1^st^ day of 5^th^ instar to 1^st^ day of eclosion). Total RNA from the wing discs and wings were extracted using the EZ.N.A^TM^ Microelute Total RNA Kit (Omega), and reverse transcribed into cDNA using oligo (dT) primer and M-MLV reverse transcriptase (Promega) according to the manufacturer’s protocol. The analysis of spatial gene expression profile consisted of various tissues from the 3^rd^ day of 5^th^ instar *Dazao* strain including wing disc, anterior silk gland, middle silk gland, midgut, malpighian tubule, fat body, gonad, head, nerve, integument, hemocyte, and trachea. The *Bombyx mori eucaryotic translation initiation factor 4 A* (microarray ID: sw22934) was used as an internal control. All experiments were performed in three independent biological sets and results were presented as mean ± S.D. Statistical analyses were performed using Student’s *t*-test (n = 3), and *P* values less than 0.05 were considered statistically significant. Primers used in qRT-PCR are listed in Table S1.

### RNAi

The ds*BmLanB1-w and* ds*Red* (red fluorescent protein, used as control) were synthesized using RiboMAX^TM^ Large Scale RNA Production System-T7 kit (Promega). Based on the developing periods of wings and the *BmLanB1-w* temporal expression pattern, we performed RNAi injections two times to each individual: first injection was in the 1^st^ hour after wandering initiated and the second was 24 h after the start of wandering. Each injection dose was 60 μg/individual. Phenotypes were observed 6 h after pupation and expression level of *BmLanB1-w* was analyzed by qRT-PCR.

### Homology search and Phylogenetic Analysis

Homologs of α, β, and γ subunits in other silkworm species were identified in SilkDB using TBLASTN and BLASTP searches. We also performed TBLASTN and BLASTP searches in the genomes of *C. elegans* and five other insects (*P. xylostella*, *H. melpomene*, *D. plexippus*, *T. castaneum*, *and A. mellifera)* to identify laminin genes. The amino acid sequences of predicted laminin genes in the related genome were consistent with the laminin proteins in GenBank (http://www.ncbi.nih.gov/GenBank/). Therefore, we downloaded the amino acid sequences of the laminin α subunit (LanA, Lanα1,2), laminin β subunit (LanB1), and laminin γ subunit (LanB2) of these species from GenBank. Phylogenetic trees were constructed by the Maximum likelihood method using MEGA6^24^ with the default settings. To evaluate branch strength in the phylogenetic tree, a bootstrap analysis of 1000 replicates was performed.

## Additional Information

**How to cite this article**: Tong, X. *et al.* A novel laminin β gene *BmLanB1-w* regulates wing-specific cell adhesion in silkworm, *Bombyx mori*. *Sci. Rep.* **5**, 12562; doi: 10.1038/srep12562 (2015).

## Supplementary Material

Supplementary Information

Supplementary Dataset
