# Supplementary material for: A novel laminin β gene BmLanB1-w regulates wing-specific cell adhesion in silkworm, Bombyx mori
Source: Sci Rep. 2015 Jul 27;5:12562. doi: 10.1038/srep12562 (PMC4515764; doi:10.1038/srep12562)
Supplement: Supplementary Information [file srep12562-s1.pdf]

Supplementary information

A novel laminin β gene *BmLanB1-w* regulates wing-specific cell adhesion in silkworm, *Bombyx mori*

Xiaoling Tong*, Songzhen He*, Jun Chen, Hai Hu, Zhonghuai Xiang, Cheng Lu and Fangyin Dai

State Key Laboratory of Silkworm Genome Biology, Key Laboratory for Sericulture Functional Genomics and Biotechnology of Agricultural Ministry, Southwest University, Chongqing, 400700, China

*These authors contributed equally to this work.

Corresponding authors:

State Key Laboratory of Silkworm Genome Biology, Key Laboratory for Sericulture Functional Genomics and Biotechnology of Agricultural Ministry, Southwest University, No. 1 Tiansheng Rd., Beibei District, Chongqing, 400700, China.

E-mail: fydai@swu.edu.cn; lucheng@swu.edu.cn

2 Supplementary Figures

2 Supplementary Tables

Supplementary Figure legends

Figure S1 - Numbers of laminins in various species. We identified laminin genes in *Caenorhabditis elegans*, *Bombyx mori*, *Plutella xylostella*, *Heliconius melpomene*, *Danaus plexippus*, *Tribolium castaneum*, and *Apis mellifera*. Numbers of laminins in *Homo sapiens, Mus musculus,* and *Drosophila melanogaster* are listed based on previous studies [8, 26]. Note that the numbers of laminins are different among the four lepidopterans. An extra α chain is present in *P. xylostella* and an additional β chain is observed in silkworm.

Figure S2 - Phylogenetic analysis of laminin β in various species. Maximum likelihood tree was constructed based on the amino acid sequences of laminin β. Two distinct orthologous groups are observed in the tree representing insects and vertebrates, respectively. Abbreviations, Bm: *Bombyx mori*, Dm: *Drosophila melanogaster*, Am: *Apis mellifera*, Aa: *Aedes aegypti*, Tc: *Tribolium castaneum*, Hs: *Homo sapiens*, Dr: *Danio rerio*, Ce: *Caenorhabditis elegans*. Accession NO.s: HsLanβ1 (NP_002282.2), HsLanβ2 (NP_002283.3), HsLanβ3 (NP_000219.2), HsLanβ4 (NP_031382.2), DrLanβ1a (NP_775382.1), DrLanβ1b (NP_001264059.1), DrLanβ2 (NP_001229974.1), DrLanβ2l (XP_692838.6), DrLanβ3 (XP_700808.5), DrLanβ4 (NP_775383.1), AmLanB1 (XP_001122457.2), TcLanB1 (XP_008199694.1), DmLanB1 (NP_476618.1), AaLanB1 (XP_001657107.1), CeLanB1 (NP_500734.2).

Figure S1.

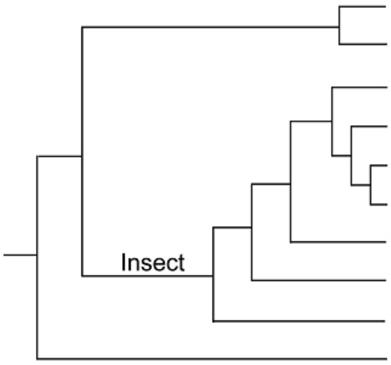

chains / species	laminin-α	laminin-β	laminin-γ
Homo sapiens	5	4	3
Mus musculus	5	3	3
Plutella xylostella	3	1	1
Bombyx mori	2	2	1
Danaus plexippus	2	1	1
Heliconius melpomene	2	1	1
Drosophila melanogaster	2	1	1
Tribolium castaneum	2	1	1
Apis Mellifera	2	1	1
Caenorhabditis elegans	2	1	1

Figure S2.

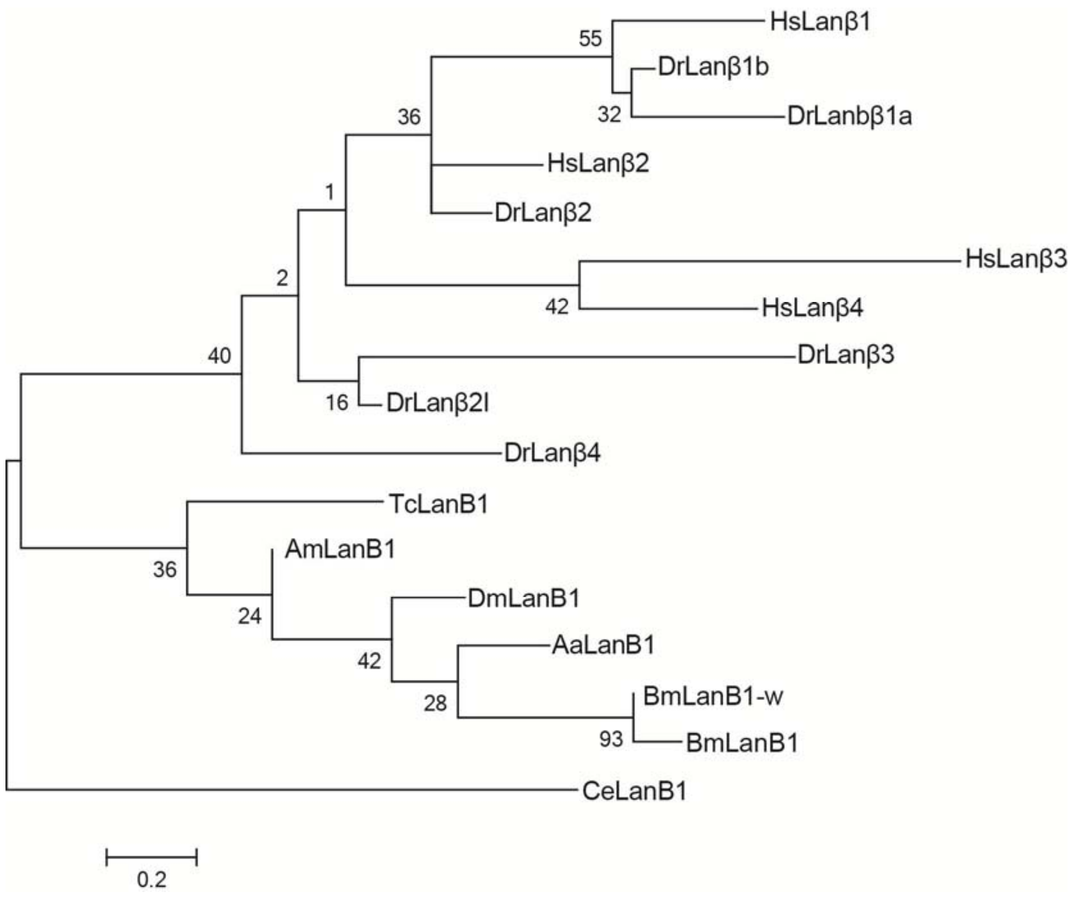
HsLanβ1
55
DrLanβ1b
32
DrLanbβ1a
36
HsLanβ2
1
DrLanβ2
HsLanβ3
2
42
HsLanβ4
40
DrLanβ3
16
DrLanβ2l
DrLanβ4
TcLanB1
AmLanB1
36
DmLanB1
24
AaLanB1
42
BmLanB1-w
28
93
BmLanB1
CeLanB1
0.2

Table S1 –The primers used in this work.

Object	Name	Sequence (5'–3')
**Linkage analysis**	S2213-F	GCCTTTATTAACGGATGCGGG
	S2213-R	AATGAGCGATTTAGTGGGCAA
	S1309-F	TTACAACTTTGAGATGCGATTTT
	S1309-R	GCAGATTTAATCGGCGTTCCC
	S1311-F	ACTACCCGTAGTTGTAATTTGATCTTTT
	S1311-R	ACGTGTTTGGTTTGCTCGCT
	M1-F	CTCTAGCAAGAGCGGTGGTT
	M1-R	ACTCTGCCTTACCTTCTTCGT
	M2-F	CTCTGAAGACATTCCGCTAA
	M2-R	TACGACAATGACGCAAGG
	M000915-F	GTGCTGGCGTTGTGATTAG
	M000915-R	CCACTTCCTGGGCTGACT
**primers for cloning**	5'RACE-GSP	GTATCGGTAGGTCCGCCAGGTTTTT
	5'RACE-nestGSP	GCAGAGTAGCGTTGGTAGAATCG
	3'RACE-GSP	ACGAGGCTCAAGCAGCACAAGATG
	3'RACE-nestGSP	ACAAATGTTGCTGGACCGTGCTAC
	P1 -F	AGAGACTACACCGCAGTATGAT
	P1 -R	AAAGCCCAAGCGAGGA
	P2 -F	GGGTGGGTCATTAGACCACG
	P2 -R	TGAGGAGATAATGAGCCTACGG
	P3-F	GAATACCGCTGGAGATAGTTG
	P3-R	GTGCCCGTTCCCTTGA
	P4-F	CTTGAAGCCCTACGATTACGA
	P4-R	GCTACATACACAACATACATACGC
**Quantitative RT–PCR**	BmLanB1-wQ-F	CGAGGCTCAAGCAGCACAAGA
	BmLanB1-wQ-R	CGGTAAATGCGAAAGTCGTAATC
	BGIBMGA002018Q-F	CAACTGCGGCGGTGAGAC
	BGIBMGA002018Q-R	CCCATAAACGGCTGGTAGAAG

	BGIBMGA000910Q-F	TGGAACTGGATGTGACCCTTG
	BGIBMGA000910Q-R	TTGTGTATGCTCCTGTGGCG
	BGIBMGA002114Q-F	CAATGAATCCAGGCTTCTCGC
	BGIBMGA002114Q-R	TCGGCTATCAAACGGTCCAG
	BGIBMGA001218Q-F	GGTATTCCCCGCTTTTCACG
	BGIBMGA001218Q-R	CAGGTGTGGGCTGGACATCTT
	sw22934 -F	TTCGTACTGGCTCTTCTCGT
	sw22934 -R	CAAAGTTGATAGCAATTCCCT
**RNAi**	BmLanB1-wdsRNA1-F	TAATACGACTCACTATAGGGAGAATCTGCCAGTAATAATGCCAATC[illegible]
	BmLanB1-wdsRNA1-R	TAATACGACTCACTATAGGGAGAGCTTCGTAGCACGGTCCAG
	BmLanB1-wdsRNA2-F	TAATACGACTCACTATAGGGAGAAGCGGAACTCAAAGCAAGA
	BmLanB1-wdsRNA2-R	TAATACGACTCACTATAGGGAGACGAAAAACATTTAAGGGCAT
	BmLanB1-wdsRNA3-F	TAATACGACTCACTATAGGGAGAGTTGAAGCCCTACGATTACGA
	BmLanB1-wdsRNA3-R	TAATACGACTCACTATAGGGAGAGCACAATATCTGTAGTTCTCGGA[illegible]

Table S2 – Abbreviation table of proteins used in phylogenetic trees

Abbreviation	Species	Subunits	Accession NO.
Dmwb	*Drosophila melanogaster*	lamininα	AAD31714
DmLanA	*Drosophila melanogaster*	lamininα	AAF50672
DmLanB1	*Drosophila melanogaster*	lamininβ	AAF52563
DmLanB2	*Drosophila melanogaster*	lamininγ	AAF50238
AmLanα1,2	*Apis mellifera*	lamininα	XP_006571726
AmLanA	*Apis mellifera*	lamininα	XP_396118
AmLanB1	*Apis mellifera*	lamininβ	XP_001122457
AmLanB2	*Apis mellifera*	lamininγ	XP_003249128
AaLanα1,2	*Aedes aegypti*	lamininα	XP_001653373
AaLanA	*Aedes aegypti*	lamininα	XP_001659481
AaLanB1	*Aedes aegypti*	lamininβ	XP_001657107
AaLanB2	*Aedes aegypti*	lamininγ	XP_001650503
TcLanα1,2	*Tribolium castaneum*	lamininα	XP_008194088
TcLanA	*Tribolium castaneum*	lamininα	XP_972827
TcLanB1	*Tribolium castaneum*	lamininβ	EFA11219
TcLanB2	*Tribolium castaneum*	lamininγ	XP_968632
HsLanα2	*Homo sapiens*	lamininα	XP_005267038
HsLanα3	*Homo sapiens*	lamininα	NP_001121189
HsLanB1	*Homo sapiens*	lamininβ	AAA59482
HsLanB2	*Homo sapiens*	lamininγ	AAA59492
CeLanA	*Caenorhabditis elegans*	lamininα	BAA32347
CeLanB1	*Caenorhabditis elegans*	lamininβ	NP_500734
CeLanB2	*Caenorhabditis elegans*	lamininγ	NP_509204
